# Information for Persons Desirous of Entering the Medical Staff of the Army

**Published:** 1861-11

**Authors:** 


### INFORMATION FOR PERSONS DESIROUS OF ENTERING THE MEDICAL STAFF OF THE ARMY.

No person can receive the appointment of Assistant Surgeon in the Army of the United States, unless he shall have been examined and approved by an Army Medical Board, to consist of not less than three Surgeons or Assistant Surgeons, to be designated for that purpose by the Secretary of War; nor can any person receive the appointment of Surgeon in the Army of the United States, unless he shall have served five years as an Assistant Surgeon, and unless, also, he shall have been examined by an Army Medical Board, constituted as aforesaid.

Boards of Medical Examiners are convened at such times as the wants of the service render it necessary, when selections are made by the Secretary of War of the number of applicants to be examined for appointment of Assistant Surgeon. To the persons thus selected, invitations are given to present themselves to the Board for examination. These invitations state the time and place of meeting of the Board.

*Applicants must be between twenty one and thirty years of age.* The Board will scrutinize rigidly the moral habits, professional acquirements, and physical qualifications of the candidates, and report favorably in no case admitting of a reasonable doubt.

The Board will report the respective merits of the candidates in the several branches of the examination, and their relative merit from the whole; agreeably whereto, if vacancies happen within two years thereafter, they will receive appointments and take rank in the Medical Corps.

An Applicant failing at one examination, may be allowed a second, after two years; but never a third.

Applications must be addressed to the Secretary of War; must state the residence of the applicant, and the date and place of his birth. They must also be accompanied *(references will receive no attention)* by respectable testimonials of his possessing the moral and physical qualifications requisite for filling creditably the responsible station, and for performing ably the arduous and active duties of an officer of the Medical Staff.

No allowance is made for the expenses of persons undergoing these examinations, as they are indispensable pre-requisites to appointment; but those who are approved and receive appointments, will be entitled to transportation on obeying their first order.

The pay and emoluments of Surgeons and Assistant Surgeons are as follows: *Assistant Surgeon,* under five years' service, $53.33 per month. Rations, per month, $36; forage allowed for one horse, $8 per month. One servant, $12 per month; clothing for servant $2.50 per month. Servant's rations, $9 per month. Total amount for servant $23.50 per month. Total amount receivable, $120.83 per month.

*Assistant Surgeon,* over five years' service, $70 per month. Rations per month, $36. Forage for one horse, $8 per month. One servant, $12 per month. Clothing for servant, $2.50 month. Rations for servant, $9 per month. Total amount for servant, $23.50 per month. Total amount receivable, $137.50 per month.

Total amount receivable by Assistant Surgeon, of over ten years' service, $173.50 per month.

*Surgeon under ten years' service*, $80 per month. Rations, $36 per month. Total amount for servant, $47. Aggregate amount receivable, $187 per month. This includes the allowance for horses and servants.

*Surgeon over ten years' service*, $80 per month. Rations, $72 per month. Total amount for servants, $47 per month. Aggregate amount receivable, $223 per month.

The allowance for forage and servants is only paid to the Surgeons and Assistant Surgeons when they actually employ and keep in service the number of servants and horses charged for.

In addition to the above, Surgeons and Assistant Surgeons are allowed an additional ration per day, after the termination of every five years' service.

*War Department, January,* 1860.

